# CO_2_-Free On-Stage Incubator for Live Cell Imaging of Cholangiocarcinoma Cell Migration on Microfluidic Device

**DOI:** 10.3390/mps7050069

**Published:** 2024-09-04

**Authors:** Shahab Ud Din, Puey Ounjai, Arthit Chairoungdua, Werasak Surareungchai

**Affiliations:** 1 Nanoscience & Nanotechnology Graduate Program, Faculty of Science, King Mongkut’s University of Technology Thonburi, Bangkok 10140, Thailand; shahab.uddin@mail.kmutt.ac.th; 2 Department of Biology, Faculty of Science, Mahidol University, Bangkok 10400, Thailand; puey.oun@mahidol.edu; 3 Center of Excellence on Environmental Health and Toxicology, Office of Higher Education Commission, Ministry of Education, Bangkok 10400, Thailand; 4 Department of Physiology, Faculty of Science, Mahidol University, Rama VI Road, Bangkok 10400, Thailand; 5 School of Bioresources and Technology, King Mongkut’s University of Technology Thonburi, Bangkok 10150, Thailand; 6 Analytical Sciences and National Doping Test Institute, Mahidol University, Bangkok 10400, Thailand

**Keywords:** live-cell imaging, on-stage incubator, cholangiocarcinoma (CCA), cell migration, microfluidics

## Abstract

Long-term live cell imaging requires sophisticated and fully automated commercial-stage incubators equipped with specified inverted microscopes to regulate temperature, CO_2_ content, and humidity. In this study, we present a CO_2_-free on-stage incubator specifically designed for use across various cell culture platforms, enabling live cell imaging applications. A simple and transparent incubator was fabricated from acrylic sheets to be easily placed on the stages of most inverted microscopes. We successfully performed live-cell imaging of cholangiocarcinoma (CCA) cells and HeLa cell dynamics in both 2D and 3D microenvironments over three days. We also analyzed directed cell migration under high serum induction within a microfluidic device. Interesting phenomena such as “whole-colony migration”, “novel type of collective cell migration” and “colony formation during cell and colony migration” are reported here for the first time, to the best of our knowledge. These phenomena may improve our understanding of the nature of cell migration and cancer metastasis.

## 1. Introduction

A cell incubator is widely considered one of the most important and in-demand pieces of equipment in cell culture laboratories, enabling cell microenvironmental conditions such as temperature, carbon dioxide content, and relative humidity to provide optimal conditions for cell growth. To achieve the optimal conditions for mammalian cell culture, incubators are kept constant at 37 °C with 5% carbon dioxide, and humidity is kept at about 95% [1].

The live cell imaging of cellular dynamics, e.g., cell–cell interaction, cell–microenvironment interaction, cell division, cell motility, and cytotoxic assays was performed in the past using inverted microscopes equipped with commercial on-stage incubators [2,3,4]. These incubators are sophisticated and costly, hindering the research activities of low-budget researchers [5]. Some approaches for live cell imaging have been followed by placing the whole inverted microscope inside the cell incubators [6,7,8]. Other methods have involved open-dish incubators to control the temperature [9]. Walzik et al. developed a portable system for live cell imaging with temperature, humidity, and carbon dioxide controllers [10]. Desai et al. developed an automated cell incubation system to maintain physiological conditions [11]. The typical prices of these on-stage incubators range from USD 400 to 2000 depending on the add-ons like CO_2_, and humidity controls. An on-stage incubation system conjugated to a microfluidic device has also been reported for mammalian cell culture [12]. A polydimethylsiloxane (PDMS)-based incubator free chamber integrated with multi-electrode arrays (MEAs) was fabricated to record neural networks at sub-cellular levels [13].

Recently, live cell imaging on a stage incubator has been employed on microfluidic devices to investigate cell kinetics including angiogenesis [14], proliferation [15], wound healing, chemotaxis, and the simulation of the flow effects on the transport of molecules secreted by single cells in in vivo microenvironments [16]. Most cell processes are developmental and time-dependent, including cell differentiation, migration, apoptosis, and cell signaling, and their morphological re-shaping can be determined by live-cell imaging. Cell processes are random, and their progression occurs over a broad span, from hours to days and weeks [17]. An Arduino-based stage incubator, easily mountable on an inverted microscope, has been reported to study the effect of drug molecules on cells over a long period on a Petri dish platform [1]. In another study, a ring-shaped culture platform was used for long-term live cell imaging to investigate cell migration and migration speed using an M4 Holomonitor [3]. Yu et al. also reported a method and device for a long-term three-dimensional hepatocyte culture for maintaining cell viability over 2 weeks. However, the image quality was poor, and no evidence was provided related to the cell dynamics, such as migration or proliferation [18]. To assess the cell progression under treated conditions with an antimitotic drug, i.e., paclitaxel, mitotically arrested cells were visualized to determine whether they die from mitosis or slip back into interphase in a six-well plate microenvironment [19].

Hence, all previous on-stage incubators have been designed for a specified purpose. In our case, we are interested in determining cancer cell migration over long periods on a microfluidics device bio-mimicked to be close to capillary levels, and other cell culture platforms. The innovation and motivation for fabricating the incubator was to enhance live cell imaging, particularly for studying cell dynamics. Additionally, the incubator was designed to be cost-effective, making it accessible for laboratories with limited budgets.

Here we report a simple, easy-to-fabricate, and cost-effective on-stage incubator for live cell imaging with a high-quality image-acquisition system for cell studies on a microfluidic system. We have developed a portable on-stage incubator for the live cell imaging of Hela and CCA cell migration in 2D and 3D microenvironments on multiple cell culture platforms ranging from Petri dishes to tissue culture flasks, multi-well plates and microfluidics with channels close to capillary size. The incubator design and fabrication were carried out to ensure low fabrication and operational costs. User-friendliness, temperature stability, ease of maintenance, image quality, and sample transparency were also considered. To enhance the imaging time in a close-to-capillary-sized microfluidic device, blocking methods were introduced to achieve a maximum of >16 h of live cell imaging with a total medium volume of 150 nL.

## 2. Material and Methods

### 2.1. Stage Incubator Design, Fabrication and Imaging System

**Figure 1:**
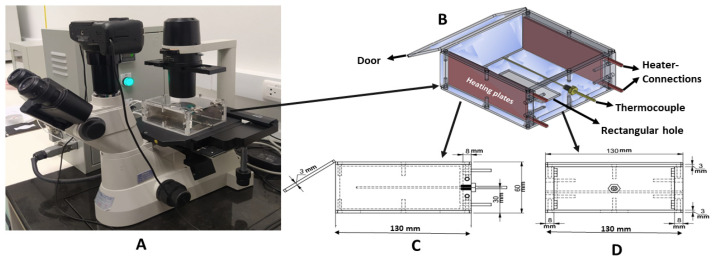
(**A**) Inverted microscope mounted with stage incubator and camera; (**B**) stage incubator design; (**C**) side view; and (**D**) front view of the incubator.

To match the stage size of our inverted microscope (Nikon eclipse TS100, Parale Mitsui Bldg., 8, Higashida-cho, Kawasaki-ku, Kawasaki, Kanagawa 210-0005, Japan) (Figure 1A) and to achieve the objectives of the study, a square-shaped acrylic incubator (130 mm × 130 mm) with a height of 60 mm (Figure 1C,D) was fabricated. The roof and the floor of the incubator were 3 mm thick, while the walls were 8 mm thick to provide temperature insulation. One of the walls was used as a door (Figure 1B) for the sample entrance. A rectangular aperture (Figure 1B) of 25 × 75 mm was drilled into the floor of the incubator. This design permits unobstructed light entry to the culture platform.

Two 100 W stainless mica strip heaters of the size 50 mm × 115 mm were placed on the two opposite walls providing a space of 5 mm to prevent the acrylic sheet from melting by direct contact with the heater.

A 12 cm long K-type stainless steel rod thermocouple (Figure 1B) was also installed in the incubator to detect the real-time temperature. It was further connected with the relay system in the main controller to switch the heating plates on and off, allowing for the adjustment of the required temperature. The thermocouple lay exactly in the middle of the incubator, 35 mm above the incubator floor, where the tip sensed and kept the incubator temperature constant.

For the live cell imaging system, a Canon 750D T6i camera (made in the America) was mounted on a microscope. The EOS utility camera connection software (version 3.11.0) with an additional open broadcaster software (OBS, version 24.0.1) was employed for image acquisition. Handbrake software (version 1.3.0) was used for video compression, and Wondershare Filmora and snipping tools from Windows 10 were used for data capture and editing. Imaging was also observed remotely by connecting the Canon-750D camera via WiFi to a smartphone.

### 2.2. Temperature Stability Test

To verify the temperature stability of the stage incubator at 37 °C, the temperature inside the incubator was recorded in triplicate daily with a digital thermometer for 12 days.

### 2.3. Applications of the Incubator

#### 2.3.1. Cell Cultures

Hela cells were obtained from the American Type Culture Collection (ATCC, Manassas, VA, USA). A CCA (KKU-213) cell line was obtained from the Japanese Collection of Research Bioresources (Osaka, Japan). To acclimatize the cells to a CO_2_-free incubator, they were cultured in Dulbecco’s modified Eagle medium (DMEM), buffered with HEPES (5.95 g/L) and incubated in a standard incubator (Labnet International, Inc., Edison, NJ, USA) at 37 °C for two weeks. For the control group, cells were fed with DMEM containing 10% FCS, buffered with sodium bicarbonate (NaHCO_3_), and incubated at 37 °C in a humidified 5% CO_2_ incubator (Thermo scientific Heracell Vios 160i, Waltham, MA, USA). The pH of the medium for both the treatment and the control group was adjusted to 7.4. No antibiotics nor antimycotics were added to the media.

#### 2.3.2. Cell Growth Kinetics

The CCA cell growth kinetics in the on-stage incubator were compared with those in a standard CO_2_ incubator (Thermo scientific, HERA CELL VIOS 160i) for seven days. Furthermore, two methods of cell number determination, the trypan blue dye-exclusion method and the MTT assay, were employed for the cell growth curve of the CCA cells.

Approximately 2 × 10^3^ cells from the treatment and the control group were seeded into each well of 96-well plates (Costar) in triplicate with 200 μL of growth medium buffered with HEPES for the on-stage incubator and with NaHCO_3_ for the CO_2_ incubator. The multi-well plate of the treatment group was sealed with a parafilm strip to prevent gas exchange and medium evaporation. Both groups were incubated for 24 h. After incubation, the cells were quantified every 24 h, for 7 days without changing the medium.

For the trypan blue exclusion method, light microscopic quantitation of cell viability was performed in a blind quantification manner according to the existing method [20]. For the MTT assay, the O.D. readings at λ = 570 nm were performed using a Cytation 5 Cell Imaging Multi-Mode Reader (BioTek, Winooski, VT, USA). The growth curves from both quantification methods were plotted, and the doubling times were calculated from the data obtained using the logarithmic phase of the cells’ growth curves.

### 2.4. Live Cell Imaging of Hela and CCA Cells in 2D and 3D Microenvironments

Live cell imaging was employed to study the cell dynamics of Hela and CCA cells in 2D and 3D microenvironments. For the 2D microenvironments, Hela and CCA cells were cultured in a plug seal cap T-25 cell culture flask (SPL Life Sciences, Pochon, Kyonggi-do, Republic of Korea) with a seeding concentration of 1 × 10^5^ cells/mL in the medium buffered with HEPES. The cells were incubated overnight in the CO_2_-free on-stage incubator. Then, live imaging of cell dynamics was monitored for 72 h with the imaging system mentioned above. The experiments were performed in triplicate.

To establish a 3D microenvironment, Matrigel (Corning^®^ Matrigel^®^ Matrix, Corning, NY, USA) was added to the cell suspension at a dilution of 1:40. The live cell imaging for the 3D microenvironment was carried out in the same way as the 2D microenvironment. The experiments were performed in triplicate.

### 2.5. Design and Fabrication of a Microfluidic Device

**Figure 2:**
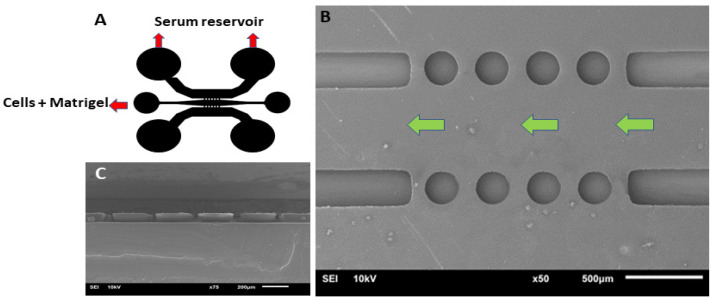
(**A**) Design illustration of the microfluidic device. (**B**) Top view of round micropillars on a silicon wafer. Green arrows show the fluidic path. (**C**) Cross-sectional view of round PDMS micropillars with scanning electron microscope (SEM).

A simple PDMS microfluidic device (Figure 2A) consisting of a channel (9.5 mm long) and 2.5 mm wide with a height of 0.1 mm was designed and fabricated using a soft-lithography technique. To achieve the containment of the Matrigel and cells, four pairs of round micropillars (250 μm diameter) for the cell’s containment with an inlet and outlet at both ends and two lateral channels with reservoirs were fabricated to enhance the stimulation medium volume (Figure 2B). The micropillars of the PDMS replicas were characterized cross-sectionally (Figure 2C) and the top view of the silicon wafer mold (Figure 2B) was characterized using a scanning electron microscope (SEM-^®^, Rock Hill, SC, USA).

CCA cells with a concentration of 300 cells/μL in DMEM medium, supplemented with 10% serum and buffered with HEPES, were mixed with Matrigel in a 40:1 ratio, respectively, at 4 °C and loaded onto the cell’s and Matrigel inlet. To induce cell migration, 100 μL of medium with 20% serum was loaded into the serum reservoirs on both sides. The experiments were performed in triplicate.

### 2.6. Medium Evaporation Reduction Methods

**Figure 3:**
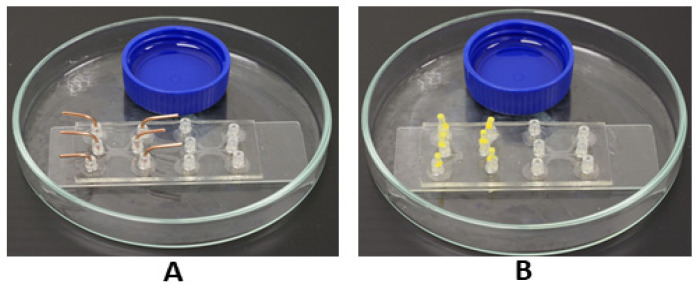
Blocking methods: (**A**) inlets of microfluidic device blocked with sterile stainless-steel pins. (**B**) Inlets of microfluidic device blocked with tips of the micropipette tips and molten wax on their top.

To enhance the imaging time in low volumes of medium, blocking methods were employed at 37 °C. Two blocking methods were used for the evaporation reduction of the medium. For the first method, sterilized stainless pins were used to insert into the inlets of the microfluidic device (Figure 3A). For the second method, the sterile tips of the 200 µL micropipette tips were cut aseptically with a scissor to insert into the inlets of the device after loading the cells (Figure 3B). The top end of the tips was then covered with molten wax to mitigate the evaporation rate. Additionally, in both blocking methods, a cap of a centrifuge tube filled with sterile water was placed in a Petri dish with the device to maintain the system humidity. Eventually, the second blocking method aided live cell imaging of CCA cell migration for almost 16 h on our CO_2_-free stage incubator.

### 2.7. Data Analysis and Live-Cell Imaging Analysis

To determine significant differences in the growth assays, *t*-test tail 1, type 1 was used, and Prism 5 was used for data visualization. Fiji image J (version 1.53m), EOS utility canon (version 3.11.0), and open broadcast streaming (OBS) were used for the image visualization of the cells. The handbrake software (version 1.3.0) was used for the video compressions, and the open-source software Wondershare Filmora (version 9.1.2) was used for the video clip editing.

## 3. Results

### 3.1. Stage Incubator Design, Fabrication and Imaging System

Our on-stage incubator (Figure 1) is lightweight (300 g), cost-effective, has a limited operational cost, and is simple to mount onto the stage of an inverted microscope, which makes it ideal for live cell imaging. The on-stage incubator also provides high image quality due to the rectangular aperture on its bottom side, which permits the unobstructed light entry into the incubator chamber. It took 12 min for the K-type mica strip heaters to reach the preset temperature of 37 °C. The temperature of the outer wall surface was 28 ± 0.6 °C, measured with the temperature sensor while the room temperature was kept at 26 °C.

### 3.2. Temperature Stability of the On-Stage Incubator

**Figure 4:**
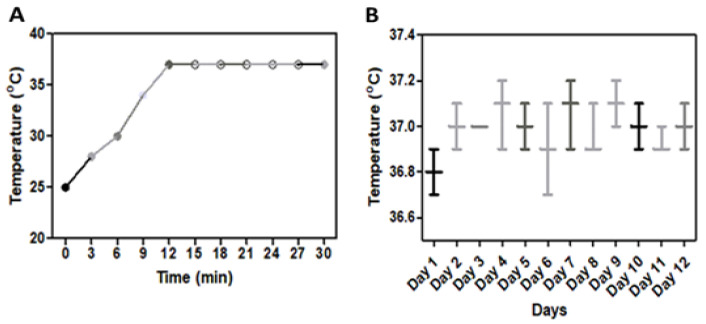
On-stage incubator temperature profile: (**A**) change in incubator temperature with time after turning on; (**B**) temperature fluctuations of the incubator over the time of 12 days.

The temperature stability of the incubator was recorded in triplicate daily with the reference thermometer (Figure 4A). It was found that the fluctuation of the temperature inside the incubator was 37 ± 0.5 °C, over 12 days (Figure 4B).

### 3.3. CCA (KKU-213) Cell Growth Kinetics in the Standard CO_2_ Incubator and CO_2_-Free On-Stage Incubator

**Figure 5:**
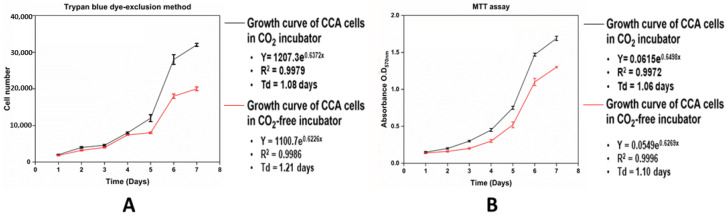
Growth curve of CCA cells in the standard CO_2_ incubator and CO_2_-free on-stage incubator: (**A**) trypan blue dye-exclusion method; (**B**) MTT assay. Error bars show ±1 standard deviation (*n* = 3). The data sets were significantly not different from day 1 to day 5 but on days 6 and 7 the data sets were significantly different, in both the trypan blue dye-exclusion method and the MTT assay for both the CO_2_ and CO_2_-free on-stage incubators. The *p*-value is <0.05 for both growth assays.

The growth kinetics of CCA cells in both the standard CO_2_ incubator and the CO_2_-free on-stage incubator were determined using the trypan blue dye-exclusion method (Figure 5A) and an MTT assay (Figure 5B). The doubling times (Td = ln × (2/B)) were calculated from the exponential slopes of the growth curves. The doubling times of CCA cells with the trypan blue method were 1.08 and 1.21 days in the standard CO_2_ incubator and the CO_2_-free on-stage incubator, respectively (Figure 5A), while the doubling times of the CCA cells with the MTT assay were 1.06 and 1.10 days in the standard CO_2_ incubator and the CO_2_-free on-stage incubator, respectively (Figure 5B).

### 3.4. Hela and CCA Cells’ Dynamics in a 2D Microenvironment

The single Hela cells migrated randomly, while the small Hela colonies were adhesive to the surface of the flask and proliferated (Video S1, recording time 38 h).

**Figure 6:**
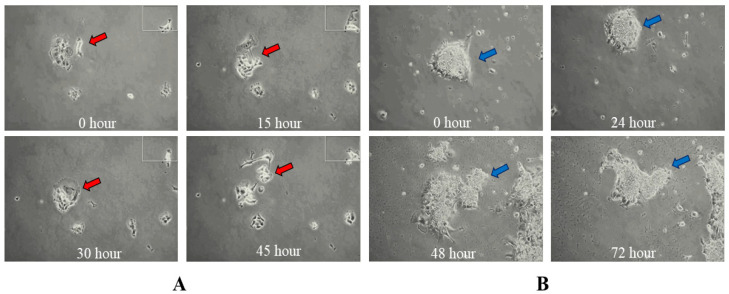
CCA colony dynamics in 2D: (**A**) CCA colony migration and dissociation; red arrow indicating the colony of interest (**B**) CCA colony migration and merging into another colony forming a bigger colony, blue arrow indicating the colony of interest. Also, see Supplementary Videos S3 and S4, respectively.

The CCA single cells and small colonies migrated randomly (Video S2, recording time 33 h). Most smaller colonies moved more actively than big colonies. Unexpected colony formation during cell and colony migration was observed. They tended to form bigger colonies and proliferated (Video S2). Moreover, at least two more interesting phenomena were discovered in a 2D microenvironment of CCA cell dynamic studies. In the first one, a novel type of collective cell migration indicated with the red arrow, each cell in the CCA colony migrated neither collectively nor coordinately but independently in random directions (Figure 6A) (Video S3, recording time = 45 h) and eventually dissociated. In the second one, the whole colony of CCA cells indicated with the blue arrow migrated in a directed manner for the first 60 h. Later, this colony merged with another bigger colony (Figure 6B) (Video S4, recording time = 72 h).

### 3.5. Hela and CCA Cells Dynamics in a 3D Microenvironment

**Figure 7:**
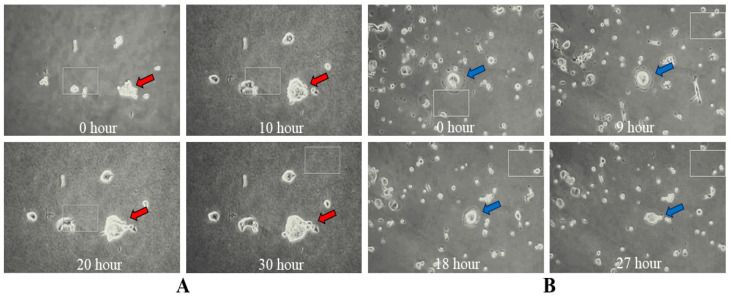
Single cell and colony dynamics in 3D microenvironment of Hela Red arrows shows the colony of interest (**A**) and CCA, blue arrow shows the colony of interest (**B**). Also, see Supplementary Videos S5 and S6, respectively.

To establish a 3D microenvironment, Matrigel (Corning^®^ Matrigel^®^ Matrix) was used at a 1:40 dilution. In the presence of Matrigel, neither Hela single cells nor colonies exhibited visible migration, unlike the monolayer Hela single cells in the 2D microenvironments (Figure 7A). Additionally, Hela colonies became more globular, adhesive, and proliferative over time (Video S5, recording time = 30 h).

However, the CCA single cells and small colonies migrated more actively and randomly in the 3D microenvironment than in the 2D microenvironment (Figure 7B) (Video S6, recording time = 27 h). Most of the single CCA cells formed small colonies initially, and then these small colonies formed bigger colonies by fusing into each other (Video S7, recording time = 35 h). Thus, the bigger colonies migrated in a slower manner, probably due to excessive attachment to the Matrigel fibers. However, some bigger CCA colonies swirled and protruded back and forth vigorously, but they did not migrate obviously over 42 h (Video S8, recording time = 42 h).

### 3.6. CCA Cell Migration in a Microfluidic Device

**Figure 8:**
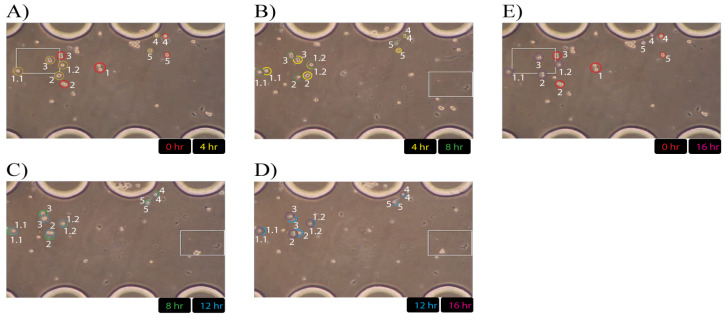
CCA cell migration in a microfluidic device: (**A**–**E**). Cells are numbered and marked with colored circles (each color represents a different image time). The circled part of the images was extracted by clipping off the rest and overlaying the extracted circles over the previous image time.

Live cell imaging of CCA cell migration within the microfluidic channel was performed over 16 h. Time-lapse images capturing the directed cell migration induced by a higher serum concentration are shown in Figure 8 (Video S9, recording time = 18 h). As soon as the cells were loaded into the loading channel, live cell imaging revealed that the cells immediately began migrating toward the stimulation reservoir containing the medium with a higher serum concentration. As shown in Video S9, the cells exhibited very active migration during the first 10 h of the experiment, while in the final 6 h, their migration rate decreased noticeably. Interestingly, we observed that the cell located near the PDMS micropillar (Cell #4) migrated significantly slower compared to the other cells in the middle of the channel, which may suggest that the hydrophobic effect of PDMS influences cell migration.

### 3.7. Blocking Methods for Medium Evaporation Reduction

**Figure 9:**
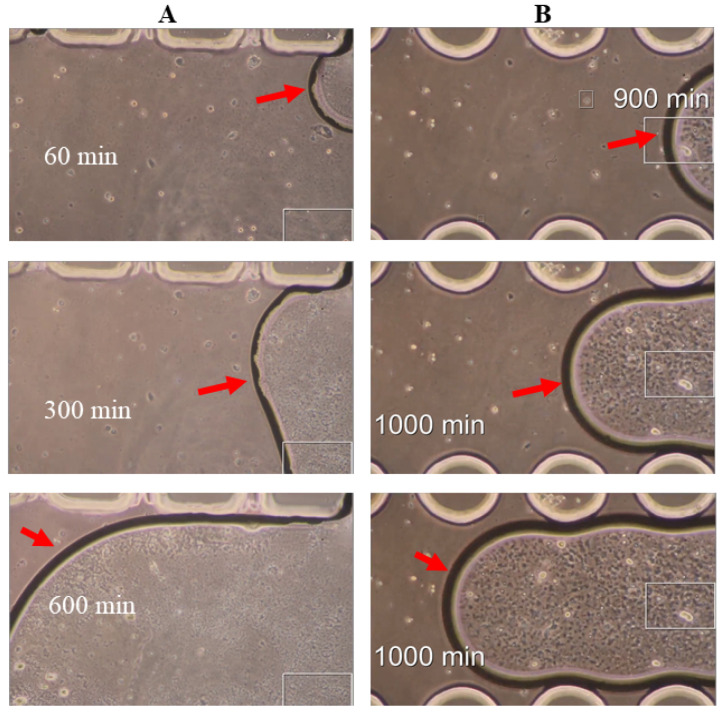
Time-lapse of medium evaporation using blocking method 1 (**A**) with sterile stainless-steel pins compared with blocking method 2 (**B**) with sterile cut tips of the 200 µL micropipette tips and filling with molten wax. The red arrows represent the meniscus of the medium evaporation with respect to time.

Medium evaporation reduction was one of the critical conditions needed to acquire long-term imaging with a limited volume on a microfluidic device. We noticed that the meniscus of the medium appeared between 3 and 4 h without a blocking method, hindering the acquisition of long-term cell migration. By employing the blocking methods, the imaging times were eventually increased by almost 4× using our relatively small volume (150 nL) of the medium. Medium evaporation was reduced two-fold by blocking method 1 (sterile stainless pins); the arrows in Figure 9A indicate the meniscus of the evaporation medium. In the case of blocking method 2 (cut tips of the micropipettes inserted in the inlets of the microfluidic device followed by covering the open ends with molten wax), the live cell imaging was almost four times as long (Figure 9B).

## 4. Discussion

The first microscope-stage incubator was devised to maintain a constant temperature, for the direct observation of the embryological development of birds [21]. Afterward, more sophisticated DIY and commercial incubators have been developed not only to control temperature but also for maintaining CO_2_ content and humidity [9,10,11,22,23].

Due to the high cost of commercial incubators, and for specific suitability, researchers used different methods of microscopic live cell observation. Some placed the whole microscope in the incubators, while others devised stage incubators for their specific purposes [24,25,26,27,28,29].

The ultimate purpose of developing our stage incubator is to study CCA cell migration and its inhibition in microfluidic devices. Thus, in this study, the on-stage incubator is a critical part of our study. Moreover, our on-stage incubator is designed in such a way that it can be applicable for multiple cell culture platforms such as Petri dishes, tissue culture flasks, multi-well plates, and in combination with other cell-monitoring platforms like microfluidics alone.

The dimensions of our incubator match the stage of an inverted microscope (Nikon Eclipse TS100). The frame of this smart incubator is fabricated with an acrylic sheet (8 mm wall thickness and 3 mm roof and floor thickness) to achieve the following properties: high transparency, high image quality, ease of installation and maintenance, insulation enhancement, light weight, and cost-effectiveness. The scratch-less nature and surface hardness of the acrylic sheet make it a suitable material compared to other polymers [30].

Image quality is enhanced due to the slide-size aperture drilled at the floor. Image quality is not affected due to the thin and transparent roof, which allows light to pass through the sample. The stainless mica strip heater is installed on both opposite walls to equally transfer heat while the tip of the thermocouple hangs exactly in the center of the incubator to detect the real-time temperature. Mica strip heaters were selected for our incubator because of the following attributes: (a) fast heat-up and response time to input—it takes only 12 min to reach the required cell culture temperature and the temperature remains constant for the rest of the experiment; (b) rust resistance; and (c) rigidity.

K-type thermocouples have several advantages that make them a popular choice for temperature measurement. One of the main advantages is their high accuracy and stability over a wide temperature range. The thermocouples can measure temperatures ranging from −200 °C to 1260 °C with an accuracy of ±2 °C or better [31]. They are also resistant to different chemicals and physical damage, making them suitable for use in harsh environments. Another advantage of these thermocouples is their low cost and easy availability. In addition, they are very easy to install and maintain, making them a convenient choice for many applications.

The temperature stability was a critical point for optimal cell growth kinetics. Regular double-checking with the reference thermometer was performed to monitor in our incubator studies. The temperature fluctuation was ±0.5 °C over a course of 12 days.

Real-time live cell imaging of Hela and CCA cell proliferation, colony expansion and migration both in single-cell and colony forms, and cell migration on microfluidics using a Canon T6i DSLR camera with our stage incubator is user-friendly. Live cell-imaging data can be transferred or uploaded in less than 5 min by pausing the imaging. This does not affect the overall time-lapse imaging. Instead of using the camera storage directly, OBS software was used additionally in combination with the EOS utility software (version 3.11.0) for longer recordings. Additionally, live cell monitoring has also been performed remotely by connecting the Canon camera to a mobile phone application (Camera Connect, version 2.9.20.18.).

As proof of durability, the incubator has been used for more than 3 years for live cell imaging. We used our incubation system continuously for as long as 7 days for cell growth kinetics, 3 days for live cell imaging of Hela and CCA cell dynamics in 2D and 3D microenvironments, and about 18 h for cell migration on microfluidics. Moreover, the incubator was utilized for the live cell imaging of cytotoxicity tests.

Medium evaporation and gas exchange issues in long-term live cell imaging were challenging in our simple incubator system. To lengthen the imaging time in a close to capillary-sized microfluidic device, blocking was introduced to achieve a maximum of >16 h of imaging with a total medium volume of 150 nL. We found that out of the two employed blocking methods, the method of using a micropipette tip blocked with molten wax enhanced the imaging time four-fold in a microfluidic device with a limited volume of 150 nL, additionally sealing the gaps of the multi-well plate with parafilm [32].

In most cell culture systems, sodium bicarbonate is used to maintain the pH [33]. However, to maintain the cancer cells in our CO_2_-free on-stage incubator, HEPES was used in a culture medium as an alternative for maintaining the pH. HEPES is a zwitterionic sulfonic acid buffer that makes its net charge zero [21]. The optimal required pH for most cell cultures is between pH 7.2 and 7.4, while HEPES has a buffering range of pH 6.8–8.2, which is therefore suitable for cell cultures. Furthermore, the stability of HEPES in maintaining pH is higher than other buffers like TRIS and PBS (phosphate buffer saline) [22]. Therefore, HEPES is employed to make the system simpler and more cost-effective.

To verify the compatibility of our on-stage incubator, we evaluated CCA cell growth kinetics in both the standard CO_2_ incubator and our CO_2_-free on-stage incubator using an MTT assay and the trypan blue dye-exclusion method. The significant differences in the growth assays were determined using a one-tailed *t*-test (type 1). Generally, CCA cell growth kinetics in both conditions followed a typical growth pattern. The growth kinetics and doubling time of CCA cells did not differ significantly between the two incubators over the first five days. However, significant differences were observed on days 6 and 7. These results were further validated by the live imaging of cell kinetics in both 2D and 3D microenvironments, recorded over 72 h (Video S4). This evidence demonstrates that our incubator is suitable for studying cell migration in microfluidic devices [34,35,36,37].

Cell migration, including both single-cell and collective-cell migration, is essential for normal physiological processes and cancer metastasis [38]. In a 2D microenvironment with a flat substrate, factors such as substrate stiffness, cell–cell interactions, cell–substrate adhesion, and chemical gradients can influence migration [29]. In a 3D microenvironment, additional variables like the matrix density and architecture, as well as the presence of collagen and other matrices, further affect both single-cell and collective-cell migration [39].

For the persistent migration often occurring in embryo development and cancer metastasis, an optimum number of cells in a small group (N < 10) was established. It was also found that large colonies (N >> 10) may dissociate into smaller groups because of cell proliferation. Furthermore, no colony breakage was found in 2D cell monolayers even in the absence of cell proliferation. According to a previous simulation [40], if the intercellular pulling forces become higher than cell–cell adhesion forces, it results in colony breakage. However, no colony breakage was observed in the case of strong adhesive forces between the cells [40].

In recent years, live cell imaging utilized sophisticated equipment to study cell metastasis has made significant advancements, establishing itself as a crucial component of tissue engineering and cancer research [41,42,43,44,45]. In our study, however, the primary objective was to investigate CCA cell migration within a microfluidic device using a simple on-stage incubator.

We conducted live cell imaging of HeLa and CCA cell kinetics within both 2D and 3D microenvironments using this incubator. During our studies in the 2D microenvironment of CCA cells, we observed several novel phenomena: a new type of collective cell migration (Figure 6A; see also Video S3), whole-colony migration (Figure 6B; see also Video S4), and colony formation during cell and colony migration (Video S2). To the best of our knowledge, these phenomena are reported here for the first time. They differ from known single and collective cell-migration behaviors [46,47,48,49,50]. Further investigation is needed to deepen our understanding of the metastatic behavior of CCA cells and cancer cells in general.

It is evident from the live cell imaging in 3D microenvironments that the Hela single cells attached more firmly to the Matrigel fibers as previously reported [51] compared with the CCA cell colonies. In the case of the Hela cell colonies, they adhered to the Matrigel fibers and proliferated similarly to a 2D microenvironment. However, they became more globular. The CCA single cells migrated actively and tended to attract each other to form increasingly bigger colonies, probably due to the higher signaling between them. As far as is known, this is the first report on the long-term live cell imaging of CCA colony formation from single cells during cell migration. The CCA colonies were able to rotate freely as well as migrate in a random direction.

As proof of the chemotaxis principle, serum-induced CCA cell migration was employed. The microfluidic device was fabricated for simple and effective operation for loading, stimulation, long-term monitoring and the measurement of cell migration. The directed migration of CCA single cells was tracked periodically for 16 h toward the serum reservoir. At first, the CCA cells migrated faster in the first 10 h and then the speed of the cell migration became slower, probably due to the diffusive factor of the serum and evaporation of the medium. To reduce the medium evaporation, the inlets were blocked by sterile 200 uL micropipette tips filled with melted wax. To make the live cell imaging effective for tracking cell migration, remote access to the microfluidic device was used for cell monitoring.

## 5. Conclusions

An on-stage incubator is essential for live cell bio-imaging. Although our incubator is simple, it is comparable with sophisticated commercial incubation systems. It is applicable for multiple cell-culture platforms including microfluidic devices coupling with electrochemical biosensing, which is a direction for future work. The transparency, image quality, ease of installation and maintenance, insulation enhancement, light weight and cost-effectiveness are favorable characteristics. Furthermore, the mica strip heaters and K-type thermocouples were selected due to their optimal functions, especially temperature stability.

Additionally, our on-stage incubator offers enhanced utility and versatility by being adaptable for integration with various inverted or stereo microscopes. Furthermore, it has been employed effectively to regulate reaction temperatures in some experimental settings.

Whole-colony migration, a novel type of collective cell migration, and colony formation during cell and colony migration were discovered during cell dynamics studies and to the best of our knowledge are reported here for the first time. These relatively simple procedures could lead to a better understanding of the sophisticated mechanisms involved in cancer progression to improve cancer survival rates.

Although this incubator has certain limitations, particularly its inability to utilize CO_2_ for pH balance, it remains suitable for conducting short-term experiments, such as those lasting up to 72 h. However, it is essential to interpret the results with the understanding that these studies were conducted in a CO_2_-free incubator, which may affect some experimental outcomes.

## Data Availability

The data presented in this study are available on request from the corresponding author.

## Supplementary Materials

The following supporting information can be downloaded at: https://www.mdpi.com/article/10.3390/mps7050069/s1: Videos S1–S9.
